# Health related quality of life in trauma patients. Data from a one-year follow up study compared with the general population

**DOI:** 10.1186/1757-7241-19-22

**Published:** 2011-04-08

**Authors:** Kirsti Tøien, Inger S Bredal, Laila Skogstad, Hilde Myhren, Øivind Ekeberg

**Affiliations:** 1 Department of Research and Development and Department of Critical Care Nursing, Division of Critical Care, Oslo University Hospital, Ulleval Hospital, PO Box 4956, Nydalen, NO-0424 Oslo, Norway; 2 Unit of Breast and Endocrine surgery, Oslo University Hospital, Ulleval Hospital, PO Box 4956, Nydalen, NO-0424 Oslo, Norway; 3 Institute of Health and Society, Faculty of Medicine, University of Oslo, PO Box 1018, Blindern, NO-0315 Oslo, Norway; 4 Department of Research and Development, Division of critical care, Oslo University Hospital, Ulleval Hospital, PO Box 4956, Nydalen, NO-0424 Oslo, Norway; 5 Department of Cardiology, Oslo University Hospital, Ulleval Hospital, PO Box 4956, Nydalen, NO-0424 Oslo, Norway; 6 Department of Acute Medicine Oslo University Hospital, Ulleval Hospital, PO Box 4956, Nydalen, NO-0424 Oslo, Norway; 7 Department of Behavioural Sciences in Medicine, Faculty of Medicine, University of Oslo, PO Box 1110, Blindern, NO-0317 Oslo, Norway

## Abstract

**Background:**

Trauma patients have impaired health-related quality of life (HRQOL) after trauma. The aim of the study was to assess HRQOL during the first year after trauma and hospital stay in trauma patients admitted to an intensive-care unit (ICU) for >24 hours compared with non-ICU trauma patients and the general population, and to identify predictors of HRQOL.

**Methods:**

A prospective one-year follow-up study of 242 trauma patients received by the trauma team of a trauma referral centre in Norway was performed. HRQOL was measured using the Medical Outcomes Study Short Form 36 (SF-36) at 3 and 12 months.

**Results:**

The mean age of the cohort was 42.3 years (95% CI, 40.4-44.3 years). The median Injury Severity Score (ISS) was 10, interquartile range 16. The HRQOL improved significantly from the 3 to the 12 months follow up in the trauma patients. However their scores were significantly lower for most subscales of SF-36 compared to the general population. Significant differences between ICU and non-ICU patients at 12 months were observed only for physical functioning and role physical subscales. Optimism was an independent predictor of good HRQOL at 12 months, in all dimensions (beta, 0.95-2.45). A higher depression score at baseline predicted lower HRQOL in four of eight dimensions (beta -1.1 to -1.70). In addition, better physical functioning was predicted by lower age (beta, -0.20), and having head injury (reference) as the most severe injury vs. spine or extremity injuries (beta, -9.49 and -10.85), and better mental health by higher age (beta, 0.21) and being employed or studying before the trauma (beta, 12.27). In addition to optimism good general health was predicted by lower score for post-traumatic stress (PTS) symptoms at baseline (beta, -0.27) and lower ISS score (beta -10.59).

**Conclusions:**

The HRQOL improved significantly from the 3 to the 12 months follow up in our sample. However their scores were significantly lower for most subscales of SF-36 compared to the general population. Significant differences between ICU and non-ICU patients were observed for only two subscales. Better HRQOL at 12 months was predicted mainly by optimism, low score for depression and PTS symptoms at baseline. High ISS predicted low general health exclusively.

## Background

As trauma care has improved substantially during recent decades and has led to higher survival rates [1], there has also been an increasing focus on the patients' perceived health-related quality of life (HRQOL) as an outcome after trauma [2]. There is growing evidence that trauma patients have impaired HRQOL after trauma [3-6] compared with reported pre-injury levels and with HRQOL in general populations [5-7]; however, the majority of this evidence stems from patients with serious injuries (Injury Severity Score (ISS) > 15) [4,8-15]. Although patients with minor injuries (ISS < 9) contribute to a large part of the health burden among adults [16], there is less documentation regarding the impact of minor injuries on HRQOL and, in particular, few studies have been performed in populations with the whole range of injury severity [5,17-19]. Studying a mixed trauma population with different levels of injury severity provides an opportunity to investigate the impact of the relative contribution of physical and mental factors to HRQOL.

Increased knowledge of HRQOL predictors after trauma may enable us to optimize and individually tailor interventions at an early stage in treatment and rehabilitaiton. The predictors of good HRQOL after trauma previously reported include lower age [4,9,20], male gender [21,22], absence of pre-existing disease [3,6,20], lower ISS [15,20,23-25], lower number of injuries [17], absence of hip/lower extremity fracture or spine injury [18], short hospital stay [17], not having been admitted to an ICU [17] and absence of head injury [6,15,20]. Other reported predictors are low post-traumatic stress [4,26] and depression scores [4]. Only three studies examined the impact of psychological distress on HRQOL after injury and hospital stay in a mixed-trauma population [5,19,27]. Two of them reported that psychiatric morbidity (mainly post-traumatic stress disorder (PTSD) and depression) predicted worse HRQOL after injury [5,27]. In contrast, one study [19] reported that anxiety shortly after injury was a predictor of better physical health. To assess the relative contribution of physical and mental factors after trauma to HRQOL, it is also important to investigate the impact of psychological distress in these patients.

Although several studies have shown that ICU patients have reduced quality of life after the ICU stay [28,29], it is not clear whether the ICU stay adds to the burden of trauma and whether the trauma ICU patients consequently report lower HRQOL than the non ICU-patients. To the best of our knowledge, few previous studies have investigated whether trauma patients who required ICU treatment had poorer HRQOL compared to hospitalized patients who did not require it.

General life orientation (pessimism vs. optimism) has in previous studies shown to be a predictor of HRQOL in ICU patients [29], depression in trauma ICU patients [30], and psychological morbidity in breast cancer patients [31]. We are not aware of any previous studies that have investigated the impact of general life orientation on HRQOL in trauma patients.

To fill in these gaps in knowledge, we performed a study with the following aims.

• Assess health-related quality of life during the first year after trauma and hospital stay and compare it with scores from the Norwegian general population.

• Compare health-related quality of life in trauma patients who required intensive care and trauma patients who did not require intensive-care treatment.

• Identify predictors of health-related quality of life after trauma and hospital stay among demographic data, trauma characteristics, clinical and psychological variables.

## Methods

A prospective cohort study of hospitalized trauma patients with different levels of injury was performed. Trauma patients were consecutively enrolled at the Oslo University Hospital, Ulleval, from June 2005 to December 2006. This hospital is a trauma referral centre for Eastern and Southern Norway and serves a population of approximately 2.5 million people. Patients are transferred to local hospitals when specialized trauma care is no longer needed.

All patients aged between 18 and 75 years who were admitted to the hospital and received by the trauma team were eligible for inclusion. The following patients were excluded from the study: Patients visiting from abroad, patients with self-inflicted injuries, severe head injury causing cognitive impairment influencing the ability to answer a questionnaire, inability to read or understand Norwegian, unknown address or previous diagnosed serious psychiatric disorders. The ability to answer the questionnaire was assessed by a nurse at the ward or rehabilitation institution the patient was transferred to after the ICU stay. If the patient was assessed unable to answer within two months after the injury the patient was excluded from the study.

Data were recorded concerning which units the patients were admitted to and the duration of their stay. Patients with a more than 24 hour stay in ICU or recovery unit were categorized as ICU patients in the study.

After transfer from the emergency department/ICU to a ward or discharge from the hospital, eligible patients received written information regarding the study and were asked to participate. Patients provided written consent and answered a questionnaire by mail after discharge. For the ICU patients the median time from injury to first assessment was 44 days (interquartile range43 days), and for the non-ICU patients 17 days (interquartile range 20 days). Assessments were also performed 3 and 12 months later. Patients who were mailed a request about participation in the study were contacted later by telephone, to confirm that they had received the letter and to inquire whether they had questions regarding the project. One reminder was sent by mail.

### Measures

To achieve the aims of the study the following instruments were used:

The Medical Outcomes Study 36-Item Short-Form Health Survey (SF-36) was used to measure HRQOL [32]. SF-36 is a generic 36-item questionnaire with eight dimensions measuring physical functioning, role limitations because of physical problems, bodily pain, general health perceptions, vitality social functioning, role limitations because of emotional problems and general mental health. In addition, one item (health transition) measures perceived changes in health during the past year [32]. The values of each sub score are computed into a scale from 0 to 100, with higher scores indicating better functioning or freedom from pain [33].

The Norwegian translation has been validated in patients with rheumatoid arthritis [34]. It was confirmed as valid for the measurement of changes in patients with injuries [35] and is recommended to measure HRQOL in trauma patients [36]. Data available from the Norwegian normal population [37] were used to compare the scores on each dimension. At the first measurement point, only three dimensions of the SF-36 were used (bodily pain, physical function and role physical), and the patients were asked to answer these questions retrospectively, as they remembered how it was before the injury. These three dimensions were chosen as retrospective measures because we assumed they were those least likely to be influenced by a recall bias.

The Life Orientation Test-Revised (LOT-R) measures life orientation (optimism/pessimism) at baseline [38]. It consists of 10 items: six target items and four fillers. Life orientation is defined as reflecting generalized positive and negative outcome expectancies, considering optimism and pessimism, respectively, as dispositional personality traits. The six target items are computed into a sum score, which range from 0 to 24, where higher scores indicate optimism and lower scores indicate pessimism.

The 15-item Impact of Event Scale (IES) measures PTS symptoms [39]. It measures intrusion using seven items and avoidance using eight items that are scored from 0 to 5, with a total score ranging from 0 to 75, higher scores indicating more PTS symptoms. The Hospital Anxiety and Depression Scale (HADS) [40] measures symptoms of anxiety and depression. It consists of 14 questions--seven questions measure anxiety and seven measure depression, each rated from 0 to 3--and it has shown good psychometric properties in different patient populations [41]. Both subscales have scores from 0 to 21.

The Abbreviated Injury Scale (AIS) and Injury Severity Score (ISS) measure the severity of injuries. The AIS classifies each injury according to body region on a scale from 1 (minor) to 6 (currently untreatable). An AIS score ≥ 3 is regarded as serious.

The ISS yields scores from 1 to 75 for the overall severity of injuries and is the sum of the square of the AIS for the three most serious injuries in different ISS body regions [42].

Because previous research has shown that the body region with the most severe injury is a better indicator of disability than ISS [43], the most serious injury was classified in accordance with the highest AIS score within the five body regions of head, spine, upper and lower extremities, face/neck and thorax/abdomen in accordance with MacKenzie and Anke [43,44]. Lacerations and superficial skin injuries were classified as external injuries. When scores were equal in two body regions, severity was classified according to the injured body region: injuries in the head were classified as the most serious, followed by the spine, the extremities, the face and neck, the thorax/abdomen, and external injuries, in accordance with MacKenzie and Anke [43,44]. ISS was recoded into a dichotomous variable with values < 9 or ≥ 9 before used in any analysis.

The level of consciousness at the time of admission to hospital was measured using the Glasgow Coma Scale (GCS), which is scored from 3 (deep unconsciousness or dead) to 15 (fully awake) based on three different behavioural responses: best motor response, best verbal response, and eye opening, each being evaluated independently. The GCS is the sum of the scores from these three responses [45].

The physical status classification system of the American Society of Anaesthesiologists (ASA score) [46] measures physical health status prior to trauma. It consists of five categories graded from Class 1 (healthy patient) to Class 5 (moribund patient who is not expected to survive for 24 h, with or without operation).

Injury-related and medical data were collected from the Trauma Registry of the hospital, where approved trauma registrars performed AIS and ISS scorings.

### Statistical methods

Statistical analyses were performed using SPSS version 15.0. Missing data on the SF-36 were replaced according to the SF-36 manual [47]. When an item was missing on the HADS and IES, missing data were replaced with the patients' mean value for each subscale. Data on categorical variables are presented as numbers and percentages, and those with ordinal scale as median. Continuous data are presented as the mean with 95% confidence interval (CI) or as the median with interquartile range, for skewed data. Independent-sample *t *tests were used to compare the mean between two groups on continuous variables, and Mann-Whitney *U *tests were performed for data that were not normally distributed. One-sample *t *test was used to compare scores for five of the eight dimensions of the SF-36 with age and gender adjusted data from the Norwegian general population. Paired sample *t *test was performed to compare scores between 3- and 12-months. A non-parametric test for related samples was performed for three dimensions with skewed distribution. To identify clinically significant differences between the mean scores for SF-36 in the general population and the 12-month scores, z-scores (the difference between the mean score in the general Norwegian population and the 12-month mean scores divided by the SD of the general population) were calculated. A score > 0.5 was regarded as a clinically significant difference [48,49]. Pearson's chi-squared tests were used to compare categorical variables. Correlation between continuous variables were analysed using Pearson's correlation coefficient.

Univariate analysis was performed by linear regression using the eight dimensions of the SF-36 as dependent variables and entering one independent variable at a time.

Variables with a p value < 0.05 were entered into the multivariate linear regression analysis, which was run with enter and was adjusted for age and gender.

Possible interactions between those variables most likely to have significant interactions were investigated.

The level of significance was set at p < 0.05.

### Ethics

The Regional Ethics Committee and the Data Inspectorate approved the study.

## Results

### Characteristics of patients

**Figure 1:**
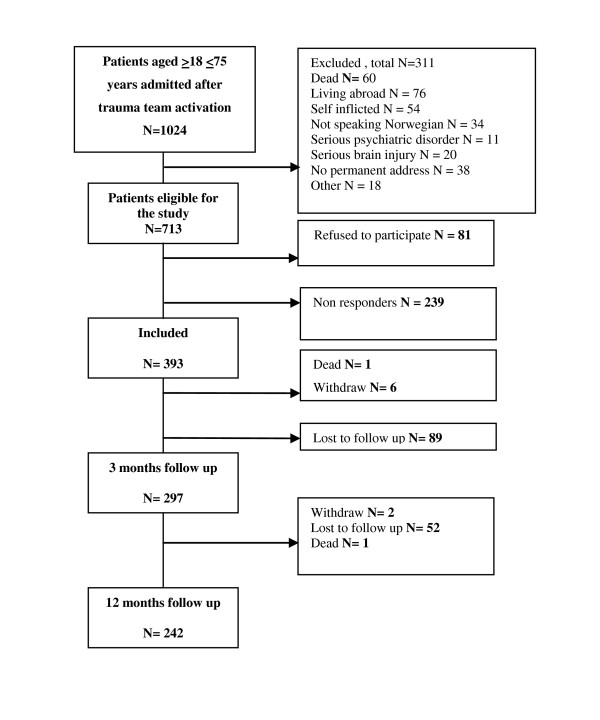
Flow chart for the study

During the recruitment period, 1,024 patients aged 18-75 years were received by the trauma team. Four hundred and fifty-six of these patients were in need of ICU treatment. Exclusion criteria excluded 34% of the ICU patients and 27% of the non-ICU patients. This difference was due to 20 ICU patients who had a severe brain injury that made them unable to answer the questionnaires. Fifty percent of the eligible ICU patients provided consent while 59% of the non-ICU patients Data for the entire sample are presented in Figure 1. The majority of patients were men (66%) and the mean age was 42.3 years (CI, 40.4-44.3 years).

Demographic and clinical variables are shown in Tables 1 and 2. A significantly larger proportion of non-ICU patients had their most serious injury (injury with highest AIS score) in the head compared to ICU patients. However, for the majority of the non-ICU patients these were not serious head injuries. Significantly more ICU patients than non-ICU patients had a *serious *head injury (AIS ≥ 3) (although this for many ICU patients not was the injury with the highest AIS score).

**Table 1:** Demographic variables for ICU patients and non-ICU patients

Demographic variables	ICU patients		Non-ICU patients		
	n = 103	%/CI	n = 139	%/CI	p
Age, mean and 95% CI^a^	42.5	39.3-45.8	42.1	39.7-44.5	0.835
Gender, male	72	71.8	86	61.9	0.105
**Level of education**					
Primary/secondary/high school	**66**	**65.3**	**70**	**52.2**	**0.044**
College or university	**35**	**34.7**	**64**	**47.8**	
**Living status**					
Married/cohabitant, yes	59	57.3	84	60.4	0.622
Caring for children, yes	**24**	**23.8**	**49**	**36.6**	**0.036**
**Occupational status**					
In work, student or retired pre injury	86	83.5	120	86.3	0.540
Out of work pre injury	17	16.5	19	13.7	
Total score IES^b^, mean and 95% CI	20.4	16.7-24.0	21.3	18.4-24.3	0.677
HADS^c ^anxiety, mean and 95% CI	5.0	4.1-5.9	6.0	5.2-6.9	0.093
HADS^d ^depression, mean and 95% CI	4.0	3.2-4.8	4.0	3.4-4.7	0.985

^a ^CI: confidence interval, ^b ^IES: Impact of event scale, baseline ^c^HADS: Hospital anxiety and depression scale, baseline

**Table 2:** Clinical variables

	ICU patients		Non-ICU patients		
	n = 103	%/CI	n = 139	%/CI	p
**Injury variables**					
Transport accident	65	63.1	97	69.8	0.275
Fall accident	22	21.4	27	19.4	0.711
Violence	5	4.9	2	1.4	^a^
Sport/leisure time/accident/working/other	11	10.7	13	9.4	0.733
**Trauma mechanism**					
Blunt trauma	100	97.1	137	98.6	0.426
Penetrating trauma	3	2.9	2	1.4	
**Most severe injury**					
Head, reference category	38	36.9	63	45.3	
Thorax/abdomen	**27**	**26.2**	**19**	**13.7**	**0.017**
Extremities	16	15.5	26	18.7	0.958
Spine	20	19.4	19	13.7	0.141
Face/external	2	1.9	12	8.6	^a^
**Injury severity**					
Minor or moderate (ISS^b ^1-8)	**4**	**3.9**	**83**	**60.1**	**< 0.001**
Serious (ISS 9-15)	24	23.3	39	28.3	
Severe (ISS 16-24)	31	30.1	13	9.4	
Critical (ISS > 24)	44	42.7	3	2.2	
Serious head injury (AIS^c ^≥ 3)	**40**	**38.8**	**11**	**7.9**	**< 0.001**
ASA^d ^score 1	78	75.7	114	82.0	0.490
ASA score 2	20	19.4	20	14.4	
ASA score 3	5	4.9	3	3.6	
GCS at arrival, mean and CI	**12.4**	**11.7-13.2**	**14.7**	**14.4-14.9**	**< 0.001**
**Length of treatment**					
Length of stay OUS^f^, days	**10.9**	**9.0-12.8**	**2.8**	**2.0-3.6**	**< 0.001**
Transferred to local hospitals	**67**	**65.0**	**29**	**20.9**	**< 0.001**
Discharged rehabilitation institution	**24**	**23.3**	**4**	**2.9**	
Discharged home	**12**	**11.7**	**106**	**76.3**	

^a^Too few to compare, ^b ^ISS: Injury severity score, ^c^AIS: Abbreviated injury scale, ^d^ASA:The American Society of Anesthesiologists Physical Status Classification, ^e^GCS: Glasgow coma scale, ^f^OUS, Oslo University Hospital Ulleval

### Non responders and dropouts

The patients who did not respond or refused to participate were significantly younger (mean age, 35.5 vs 39.3 years, p = 0.001), and a significantly greater number of these were men (77% vs 68%, p = 0.007) compared with the patients who participated and answered one or more questionnaires. Two hundred and forty-two patients answered questionnaires at all three time points. Significant differences between participants and patients who dropped out at 3 or 12 months are presented in table 3.

**Table 3:** Variables with significant differences between participants and dropouts

	Participants		Drop outs		
	n = 242	%/CI	n = 149	%/CI	p
Age, mean and 95% CI^a^	42.1	40.1 - 44.1	34.5	32.1 - 36.5	<0.001
LOT-R^b^	16.3	15.7 - 16.9	14.3	13.4 - 15.1	<0.001
Primary/secondary/high school	136	56.2	96	63.6	
College or university	99	40.9	43	28.5	0.031
Living alone	99	40.9	89	58.9	<0.001
Out of work before injury	36	14.9	38	25.5	0.009
ISS^c ^≥ 9	154	63.9	75	50.3	0.080
Injured by physical assault	7	2.9	23	15.2	<0.001
Intensive care patient	103	42.6	47	31.1	0.023
Anxiety baseline	5.6	4.9 - 6.2	7.3	6.4 - 8.2	<0.001
Total score IES^d^, mean and 95% CI	20.9	18.7 - 23.2	28.3	25.2 - 31.5	<0.001
HADS^e ^anxiety, mean and 95% CI	5.6	5.0 - 6.2	7.5	6.6 - 8.3	<0.001
HADS depression, mean and 95% CI	4.0	3.5 - 4.5	5.3	4.5 - 6.1	0.006

^a^CI: confidence interval, ^b^LOT-R: Life orientation test revised, baseline ^c^ISS: Injury severity score,^d^IES: Impact of event scale, baseline ^e^HADS: Hospital anxiety and depression scale, baseline

### Gender

The HRQOL mean value for the three dimensions measured before the injury was not significantly different between men and women. At 3 months, the only differences between men and women pertained to the dimensions of mental health (men mean, 76.6; CI, 73.7-79.5 vs. women mean, 71.3; CI, 67.2-75.4; p = 0.037) and vitality (men mean, 57.3; CI, 53.7-60.8 vs. women mean, 46.6; CI, 42.0-51.2; p < 0.001). At 12 months, gender differences were observed only for the vitality dimension (men mean, 56.8; CI, 53.2-60.5 vs. women mean, 50.0; CI, 44.6-55.3; p = 0.036). As the differences in HRQOL between men and women were negligible, the results presented were not divided according to gender. Significantly more women had minor injuries (ISS, 1-8) compared with men (51% vs. 28%; p = 0.005). The median ISS was 10, interquartilerange 16.

There was significant interaction between age, gender and all dimensions in SF-36 except bodily pain (p < 0.001). To investigate this further, age was divided on median (42 years) in the whole sample. Then differences in scores for low or high age in the seven dimensions were compared between men and women. The largest difference was in role emotional where mean score was reduced by 20 point for women when moving from low to high age but only 3 points for men. For role physical the mean score was reduced 17 points when moving from low to high age in women but only 7 points in men. For the other dimensions, these differences were more moderate. For social functioning the difference changed direction between women and men. Women reduced their score 5 points when moving from low to high age while men increased 3 points moving from low to high age.

### Health-related quality of life before injury and during first year

**Figure 2:**
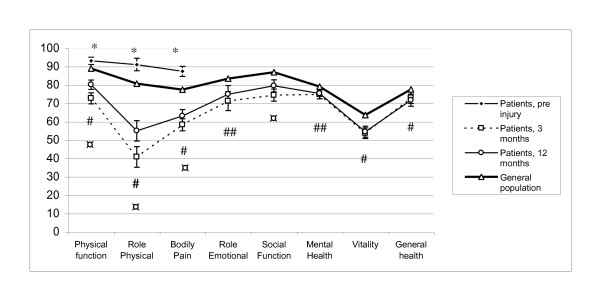
**Scores for SF-36 at 3 and 12 months compared with data from the Norwegian general population**. * Significant difference between the pre-injury score and that of the general population (p < 0.001). # Significant difference between the 12-month score and that of the general population (p < 0.001). ##Significant difference between the 12-month score and that of the general population (p < 0.05). ¤Significant improvement of the score between 3 and 12 months (p < 0.001).

The mean SF-36 scores for bodily pain, physical functioning and role physical were significantly higher before injury in patients than in the general population (Figure 2).

The improvement between 3 and 12 months was statistical significant for physical function, role physical, bodily pain and social functioning.

### Health related quality of life compared with the general population

At 12 months, the mean scores for all dimensions, with the exception of social function, were significantly lower than those of the general population. Only physical functioning, role physical functioning and bodily pain had z-scores ≥ 0.5, indicating a clinically significant difference between the general population and the scores of the patients at 12 months. The other dimensions had a z-score between 0.2 and 0.4, indicating the presence of small differences.

### Comparing ICU and non-ICU patients

**Figure 3:**
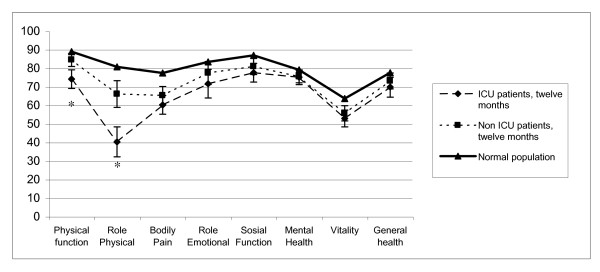
**Scores for SF-36 at 12 months for ICU patients and non-ICU patients compared with data from the Norwegian general population**. *Significantly difference between ICU and non-ICU patients; p < 0.01.

The comparison of mean scores between ICU and non-ICU patients at 12 months revealed the presence of statistically significant differences only for the physical function and role physical subscales (Figure 3).

### Predictors of health-related quality of life

The variables investigated for association with each dimension in the SF-36 were age, gender, education, living status, care of children, employed, retired or studying before the injury, ASA score before injury, GCS on admission, ISS, severe head injury (AIS ≥ 3), ICU treatment, type of accident and body region of the most serious injury, LOT, HADS depression score at baseline and PTS symptoms at baseline. Anxiety was not entered into the multivariate analyses because it was highly correlated (0.68) with the depression and IES scores. The relationship between length of stay in hospital (LOS) and HRQOL was not investigated because 40% of the patients were transferred to local hospitals. As this was a single-centre study, data on LOS in local hospitals were not available.

Good physical functioning was independently predicted by lower age, optimism (high LOT-R score), low depression score, and having head injury as the most severe injury vs spine or extremity injury (Table 4). Higher age, optimism, low depression score at baseline and being employed, studying or retired before the injury, predicted good mental health. Low ISS score, low scores for PTS symptoms at baseline and optimism were predictors of good general health. Bodily pain was predicted by PTS symptoms at baseline, depression at baseline and pessimism. Optimism and low depression cores at baseline predicted a high score for vitality. A high score for role physical function was predicted by low ISS score (beta -17.85, CI -31.17, 4.54, p = 0.009), optimism (beta, 2.45; CI, 1.16, 3.74; p = < 0.001), low depression score (beta -1.96, CI -3.61, -0.31, p = 0.020) not requiring ICU treatment (beta, -16.68, CI -29.00, -4.36, p = 0.008); (ICU treatment = 1)), Optimism and low depression scores predicted higher scores for role emotional and social functioning. In addition, good social functioning was predicted by having been employed, studying or retired before the injury, a low ISS score and low age.

**Table 4:** Multivariate linear regression analysis of five of the eight dimensions of SF-36 at 12 months

	Physical functioning			Mental health			General health			Bodily pain			Vitality		
	Beta	95% CI	p	Beta	95% CI	p	Beta	95% CI	p	Beta	95% CI	p	Beta	95% CI	p
Age	-0.20	-0.39, -0 02	0.030	0.21	0.05, 0.36	0.010									
ISS score dic^a^							-10.59	-17.28, -3.90	0.002						
LOT-R^b ^score	0.95	0.25, 1.66	0.080	1.21	0.66, 1.76	<0.001	1.74	0.95, 2.53	<0.001	0.96	0.12, 1.81	0.025	1.08	0.35, 1.80	0.004
Depression at baseline	-1.11	-2.01, -0.20	0.016	-1.44	-2.15, 0.73	<0.001				-1.23	-2.32, -0.14	0.027	-1.70	-2.64, 0.75	<0.001
IES^c ^score at baseline							-0.27	-0,49, -0.045	0.019	-0.31	-0.55, -0.07	0.011			
Working before injury^d^				12.27	5.4, 19.13	0.001									
**Most severe injury **with head as reference category															
Spine vs head	-9.49	-17.58, -1.41	0.022							-7.64	-17.23, 1.96	0.118			
Extremities vs head	-10.85	-18.74, -2.95	0.007							-5.99	-15.33, 3.35	0.207			
Thorax/abdomen vs head	0.56	-7.27, 8.39	0.888							-1.06	-10.49, 8.36	0.824			
Face/external injury vs head	4.80	-7.43, 17.04	0.440							15.4	0.61, 30.19	0.041			
Adjusted R^2^	0.24			0.38			0.28			0.27			0.29		

n = 220-225 because of missing values for some variables.

^a^ISS score dic: Injury severity score dichotomous: ISS < 9 = 0, ISS ≥ 9 = 1, ^b^LOT-R: Life orientation test revised, ^c^IES: Impact of event scale, ^d^Working before injury yes = 1, no = 0

## Discussion

The main findings in this study of trauma patients with different degrees of injury severity were that the mean scores for all subscales of HRQOL, with the exception of social functioning, were significantly lower than those observed in the Norwegian general population. Significant improvement between three and twelve months was only seen in physical functioning, role physical, pain and social functioning. Significant differences between ICU and non-ICU patients at 12 months were observed only for physical functioning and role physical subscales. Optimism was an independent predictor of good HRQOL at 12 months, in all dimensions. Other main predictors of good HRQOL were absence of depression and absence of PTS symptoms.

It might have been expected that there had been more improvement in other domains than physical functioning, role physical, pain and social functioning. On the other hand, the physical and pain domains were those most impaired, compared to the general population. Although we identified a significantly lower score in seven of eight domains at 12 months compared with that of the general Norwegian population; only physical functioning, role physical and pain had z-scores that were indicative of clinically significant differences compared with the general population.

We have identified six previous studies that compared HRQOL in trauma patients with the general population [4,7,50-52] or a healthy control group [19]; all of these studies reported significantly lower scores for the patients compared with the general population/control group but none of them presented z-scores for this difference. These six studies covered patients with pelvic ring fracture [51], mixed trauma [7,52,53], major trauma [4] and orthopaedic conditions [19] and might partly be comparable with our study.

Our patients' HRQOL scores 3 and 12 months post-trauma were significantly lower than in the general Norwegian population. In addition, our patients reported significantly higher pre-trauma HRQOL. It may therefore be argued that our patients had a greater drop in HRQOL than revealed by comparing with the general population. However, the high score before injury may well have been caused by recall bias. Watson et al. [52] also found a higher pre-injury level of HRQOL in trauma patients compared with that observed in the Australian general population. These authors compared the pre-injury level with that reported after 12 months in patients who had recovered completely from the injuries. As these two measures were similar, the authors argue that retrospectively measured pre-injury HRQOL is a valid measure of HRQOL. This might support the contention that the higher pre-injury levels of physical functioning, role physical and pain found in our study were not caused by a recall bias. One might assume that, at least for the physical dimensions, it is possible to recall these levels quite accurately. But it is also possible that our patients recall their physical and role physical functioning as better than it really was.

It might be discussed how relevant it is to compare trauma patients with the general population. The trauma population is known to have a greater proportion of people from lower social classes, with more substance abuse and criminality than the general population. Using a control group matched for income, education and occupation in addition to age and gender might have given less difference between SF-36 scores in patients and the comparing group.

Significant differences between ICU and non-ICU patients were seen only regarding physical functioning and role physical functioning at 12-months. One might have expected that ICU patients would also have lower scores in other dimensions, e.g., mental health, as it has previously been shown that ICU patients suffer from anxiety, depression and PTS symptoms after ICU stay [54,55]. We are not aware of other studies that have compared HRQOL in ICU and non-ICU patients.

Optimism was an independent predictor of good HRQOL in all dimensions. To the best of our knowledge, this is the first study to demonstrate this in a mixed-trauma population. Previous studies showed that this is a predictor of HRQOL in ICU patients [29] and coronary artery bypass patients [56]. In a study of women with breast cancer, Schou et al. found that optimistic women reported better HRQOL and that this effect was mediated by coping strategy [57]. Optimistic women used a strategy of fighting spirit, whereas pessimistic women responded with a hopeless/helpless strategy to a greater degree. Survivors of trauma have the challenge of coping with both the physical and emotional consequences of the injury. It might be that the association between life orientation and HRQOL in trauma patients is also mediated by coping strategy. To improve HRQOL, patients with a pessimistic life orientation should be identified and interventions targeting better coping should be offered. A helpless/hopeless strategy can be improved using cognitive behavioural therapy [58].

Depression at baseline was a predictor of lower HRQOL in four dimensions: physical functioning, mental health, bodily pain and vitality. As depression is a psychological symptom, it is not surprising that it predicts mental health. Lack of energy is a symptom of depression and may explain why depression at baseline predicted vitality at 12 months. More surprisingly, depression also predicted physical functioning. This might be because depressed patients have little energy, which influences their ability to exercise and to regain their previous level of function after the injury. This means that patients could be screened for depression after trauma and that depressed patients could be offered treatment for this condition. They might benefit from being taught coping strategies to manage physical training, despite the depression. We identified only two studies that investigated the association between depression and HRQOL in trauma patients. Holbrook et al.[4] reported depression as a predictor of overall lower HRQOL in major trauma patients after 12 and 18 months. Ponsford et al.[19] found that depression was a predictor of worse physical health (as measured using SF-36) in orthopaedic trauma patients with a mean ISS of 13.1. They measured depression at the same time as the outcome; therefore, it can be argued that in that study, depression was associated with physical health but was not its predictor. The injury severity score was only an independent predictor of general health. One might have expected that the severity of injury would also have an impact on other dimensions, e.g., physical functioning and role physical. It has differed between studies whether ISS has been found to be a predictor of the different dimensions in HRQOL or not. MacKenzie et al [59] and Bull [60] did not find that ISS was a significant predictor of physical functioning after injury. Vles et al.[24] found that ISS score predicted all dimensions--with the exception of anxiety and depression--of HRQOL in severely injured patients, as measured using EuroQol. Harris et al [20] also found that ISS score independently predicted the physical component score measured by SF-36 in severely injured patients, and this was also found in a mixed-trauma sample [7]. Ringdal et al.[3] and Kiely et al [61] did not find a similar result in severely injured patients and in patients with moderate-to-severe injuries, respectively. Ringdal et al. found that the APACHE II score (a measure of the seriousness of illness), which was entered into the multivariate analysis together with ISS, was an independent predictor of physical functioning. Kiely et al. used the Functional Independence Measure as an independent variable in their multivariate analysis, together with ISS, and found that it was a predictor of physical functioning. It might be that these two variables also reflect the seriousness of the injury and crowd out ISS as an independent predictor.

### Strengths and limitations

In the present study, patients were included regardless of the ISS score and of the localization of the injuries. This provided the opportunity to study the relative magnitude of the effect of mental and physical impairments on HRQOL. The response rate in this study was moderate, with a participation of 50% of the eligible ICU patients and 59% of the non-ICU patients, thus limiting the extent to which results can be generalized to the whole trauma population. The lower response rate in ICU patients compared to the non-ICU patients, might partly be caused by ICU patients having felt too weak to answer our extensive questionnaire. We might have achieved a higher response rate from the ICU patients if the questionnaire had been shorter or if they had received it later after the injury.

The survey method was chosen because many of the patients in our sample lived as far as 200-300 kilometres from the hospital and it therefore was complicated to perform interviews. When using postal surveys the patients do not have the possibility to ask questions concerning the questionnaire. To avoid this problem the patients were phoned and asked whether they had questions regarding the project and questionnaire. They also received two phone numbers they could call if they had questions. Nevertheless patients may have misunderstood some questions and this might influence the reliability of the study.

The non-responding patients were significantly younger than the responders and more of them (77%) were men. It is plausible that our results might have been different if these young men had participated.

The dropouts were more likely to have been injured by physical assault, had more mental problems and were more pessimistic. If these patients had participated, the differences in HRQOL between patients and the general population would, most likely, have been somewhat greater.

The score of the physical status classification system of the American Society of Anaesthesiologists was the only measure of pre-injury co-morbidity. In addition, we measured three dimensions of SF-36 (physical functioning, role physical function and pain) as the patients remembered them to be before the injury. We did not measure pre-injury mental problems, as a recall bias is more likely for mental states than, for example, for the assessment of the ability to walk 100 m. If more extensive information about pre injury physical and mental health had been included in the regression analysis, this might have influenced the results.

### Clinical implications

Patients could be screened for psychiatric symptoms after trauma. Patients with clinically significant depression or PTS symptoms should be offered treatment accordingly. It is plausible that these patients might be in greater need of long term follow up in physical training than those not showing significant symptoms of PTS symptoms and depression. Patients with a pessimistic life orientation should be identified and might benefit from interventions that target better coping.

## Conclusions

The HRQOL improved significantly from the three to the twelve months follow up in five of eight dimensions of SF-36 in the trauma patients in our sample. However their scores were significantly lower for most subscales of SF-36 compared to the general population.

Significant differences between ICU and non-ICU patients were observed for only two physical subscales. Better HRQOL at 12 months was predicted mainly by optimism, low score for depression and PTS symptoms at baseline. High ISS predicted low general health exclusively.

## Competing interests

The authors declare that they have no competing interests.

## Authors' contributions

KT had the main responsibility of planning the study, collecting the data, performing the data analyses and writing the article. LS also collected data. ISB, LS and HM participated in the planning of the study and discussions during data analyses, read the manuscript and participated in the general discussion of the paper. ISB made significant contributions to the manuscript by reading it and providing suggestions for its improvement. ØE participated as the main supervisor of the whole process of the study: planning of the study, analysis of the data and reading, discussion and improvement of the manuscript.

All authors have read and approved the final manuscript.
